# Intracoronary Imaging: Current Practice and Future Perspectives

**DOI:** 10.31083/j.rcm2402039

**Published:** 2023-02-02

**Authors:** Grigorios Tsigkas, Panagiota Spyropoulou, Elena Bousoula, Anastasios Apostolos, Georgios Vasilagkos, Grigorios Karamasis, Kyriakos Dimitriadis, Athanasios Moulias, Periklis Davlouros

**Affiliations:** ^1^Department of Cardiology, University Hospital of Patras, 26504 Rion-Patras, Greece; ^2^Department of Cardiology, “Tzaneio” Hospital, 18536 Athens, Greece; ^3^First Department of Cardiology, National and Kapodistrian University of Athens, “Hippocration” General Hospital Athens, 11527 Athens, Greece; ^4^Second Department of Cardiology, Medical School, National and Kapodistrian University of Athens, “Attikon” University Hospital, 12461 Athens, Greece

**Keywords:** intravascular ultrasound, optical coherence tomography, coronary artery disease, PCI guidance, co-registration, hybrid

## Abstract

Intracoronary imaging has brought new insight in the field of interventional cardiology. Intravascular ultrasound (IVUS) and optical coherence tomography (OCT) are the most commonly used imaging modalities. Regarding their technical characteristics IVUS and OCT have similarities as well as differences, a fact that could have significant clinical implications. Both techniques play an important role in percutaneous coronary intervention (PCI) guidance and demonstrated superiority compared to intravascular coronary angiography (ICA) guidance alone. Furthermore, their use can notably assist coronary plaque evaluation; both provide additional information of plaque characteristics, which can lead to a better understanding of the cause of an acute coronary syndrome (ACS) and better clinical outcomes. However, there is not enough clinical evidence for the superiority of one method compared to the other, something that is, also, reflected in the guidelines. In this review, we aim to compare role of IVUS and OCT in the different aspects of coronary artery disease (CAD), according to the latest scientific data. In addition, we present the future perspectives regarding the IVUS and OCT, with co-registration of the two methods or hybrid OCT-IVUS catheters.

## 1. Introduction

Coronary artery disease (CAD) is the leading cause of death in both high-income and middle-low-income countries [1]. The major pathophysiological pattern of CAD is coronary obstruction or compromise of blood flow due to atherosclerosis, leading to oxygen supply-demand mismatch in myocardial cells [2]. Although, invasive coronary angiography (ICA) is considered the gold standard for the diagnosis of CAD, it has some substantial limitations. Thus, it is well recognized, that ICA is not capable of assessing the full spectrum of the disease. Two are the most commonly used intracoronary imaging modalities; intravascular ultrasound (IVUS) and optical coherence tomography (OCT). Notwithstanding the established role of both methods in cardiac catheterization procedures, few studies aimed for the direct comparison of IVUS and OCT (see Table 1, Ref. [3, 4, 5, 6, 7, 8, 9, 10]) and, currently, there is no clear evidence about the superiority of one method compared to the other in different clinical settings.

**Table 1.** **Studies of OCT vs. IVUS- versus angiography-guidance in PCI**.

Trial name or first author	N	Study design	Primary endpoint(s)	Main findings
Habara *et al*. (2012) [5]	70	Randomized, OCT vs. IVUS	Post-PCI stent expansion measured by IVUS	Higher stent expansion and visualization of stent-edge plaque burden and vessel border in the IVUS group.
				No difference in stent apposition and accessibility parameters.
CLI-OPCI (2012) [3]	670	Observational, ICA plus OCT vs. ICA alone	Cardiac death or MI at 1 year	Lower rate of MACE at 1 year in the ICA plus OCT group.
				OCT led to additional interventions in 35% of patients.
ILUMIEN II (2015) [6]	940	Observational, OCT vs. IVUS	Post-PCI stent expansion measured by OCT or IVUS	Comparable degree of stent expansion in both groups.
				Stent malapposition, tissue prolapse and edge dissection more frequently detected with OCT, still no significance.
DOCTORS (2016) [4]	240	Randomized, OCT vs. ICA	Post-PCI FFR	Post-procedural FFR was greater in the OCT group.
				OCT led to additional interventions in 50% of patients.
ILUMIEN III (2016) [8]	450	Randomized, OCT vs. IVUS vs. ICA	Post-PCI MSA measured by OCT	OCT was superior to ICA and non inferior to IVUS regarding post-PCI MSA.
				OCT resulted in fewer untreated dissections and stent malappositions than IVUS.
				The EEM-based strategy for stent sizing was safe with similarly few MACE in 30 days among groups.
OPINION (2018) [7]	829	Randomized, non-inferiority, OCT vs. IVUS	TVF in 12 months	OCT was non inferior to IVUS regarding TVF in 12 months.
				MLA at 12 months was comparable in both groups. Stent sizing based on lumen diameter in the OCT group and on vessel diameter determined by the EEM in the IVUS group.
ULTIMATE (2018) [10]	1448	Randomized, IVUS vs. ICA	TVF at 12 months	Lower rate of TVF both at 12 months and 3 years in the IVUS group, especially regarding TVR.
iSIGHT (2021) [9]	151	Randomized, non-inferiority, OCT vs. IVUS vs. ICA	Post-PCI stent expansion measured by OCT	OCT was non inferior to IVUS and superior to ICA regarding stent expansion.
				Stent expansion was comparable in IVUS and ICA group.
				The EEM-based strategy for stent sizing was efficient.

OCT, Optical Coherence Tomography; IVUS, Intravascular Ultrasound; PCI, Percutaneous Coronary Intervention; CLI-OPCI, Centro per la Lotta contro l’Infarto-Optimisation of Percutaneous Coronary Intervention; ICA, Invasive Coronary Angiography; MI, Myocardial Infarction; MACE, Major Adverse Cardiovascular Events; ILUMIEN II, Observational Study of Optical Coherence Tomography [OCT] in Patients Undergoing Fractional Flow Reserve [FFR] and Percutaneous Coronary Intervention; DOCTORS, Does Optical Coherence Tomography Optimize Results of Stenting; FFR, Fractional Flow Reserve; ILUMIEN III, Optical coherence tomography compared with intravascular ultrasound and with angiography to guide coronary stent implantation; MSA, Minimum Stent Area; EEM, External Elastic Membrane; OPINION, OPtical frequency domain imaging versus INtravascular ultrasound in percutaneous coronary intervention; TVF, Target Vessel Failure; MLA, Minimum Lumen Area; ULTIMATE, Intravascular Ultrasound Versus Angiography-Guided Drug-Eluting Stent Implantation; TVR, Target Vessel Revascularization; iSIGHT, Optical Coherence Tomography Versus Intravascular Ultrasound and Angiography to Guide Percutaneous Coronary Interventions.

The aim of the current manuscript is (1) to summarize the technical characteristics of each method and the possible clinical implications of them (2) to present their distinct role on stent optimization discussing studies which compare the two techniques between them and with ICA (3) to highlight their importance in plaque evaluation and (4) to comment the future perspectives of IVUS and OCT.

## 2. History Flashback

The initial *in vitro* studies of IVUS took place in the late 1980s and showed good correlation between intravascular imaging findings and histopathological findings of the vessel wall of coronary arteries, as well as the depiction of the three-layer wall in muscular arteries [11, 12]. The first *in vivo* use of IVUS in human arteries, in the late 1980s, revealed the potential of IVUS in atheromatous plaque characterization and raised interest about the possible implications of this new modality [12].

OCT, a technique based on low-coherence interferometry, was introduced more recently. In 1991 Huang *et al*. [13] developed the OCT technique and conducted the first *ex vivo* study in two different tissues: the peripapillary part of the retina and a coronary artery. As for the coronary artery, the study delineated the ability of OCT to differentiate between healthy and abnormal tissue. The initial *ex vivo* studies revealed high correspondence of OCT findings and histopathological findings of atherosclerotic human arteries and a very detailed visualization of plaque characteristics [14]. This correspondence was, also, found in in-human studies. The first *in vivo* study was reported in 2002 by Jang *et al*. [15], demonstrating the feasibility and safety of OCT and its promising capability in better understanding of the myocardial infarction (MI) process.

## 3. Technical Characteristics & Differences

**Fig. 1.**
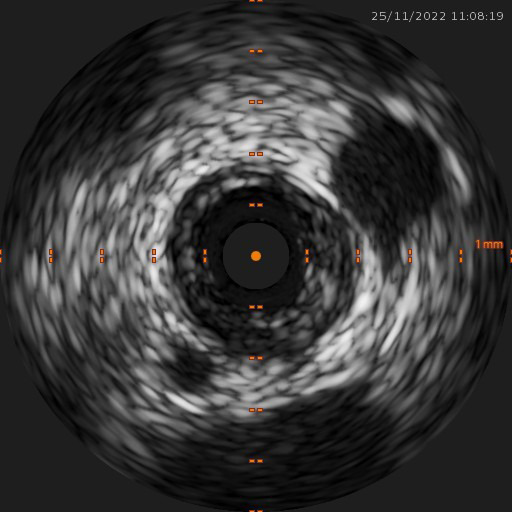
**IVUS image of a mixed fibrofatty plaque (cross sectional view). **Image from our records. IVUS, intravascular ultrasound.

**Fig. 2.**
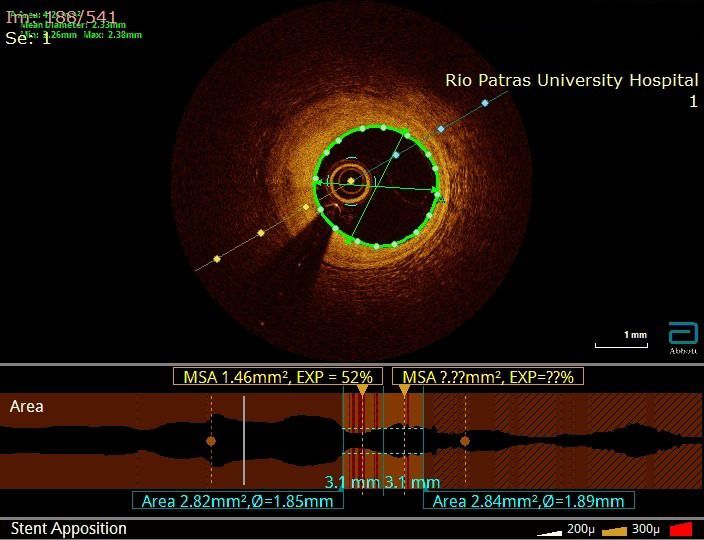
**OCT image of a fibrous plaque (cross sectional view at the upper panel, longitudinal view at the lower panel)**. Image from our records. OCT, optical coherence tomography.

OCT and IVUS provided new insight in coronary intervention enabling better understanding of the pathophysiology of CAD and giving further opportunities for its optimal treatment. Both modalities create images of intracoronary structures based on image reconstruction (Figs. 1,2). Apart from sharing similarities regarding the concept of their use, IVUS and OCT have, also, some different technical characteristics with subsequent clinical impact (Table 2, Ref. [16, 17, 18, 19, 20, 21, 22, 23, 24, 25, 26]).

**Table 2.** **Technical characteristic of IVUS and OCT and their clinical significance**.

Technical characteristic	IVUS	${\mathrm{OCT}}^{2}$	Clinical significance
Source of image	Ultrasound waves (Grey technology)	Low-coherence light (Orange technology)	Because light is attenuated by red blood cells, OCT requires blood clearance, which is achieved usually after contrast injection [16]
Sheath compatibility	5-Fr or ${\mathrm{larger}}^{1}$	5- or 6-Fr or ${\mathrm{larger}}^{2}$	Although imaging with OCT can be performed with the use of a 5-Fr sheath and guide catheter, that can produce images with lower quality, due to the need of blood clearance [17, 18]. Thus a 6-Fr or larger guide catheter is generally preferred, whereas imaging with a 5-Fr guide catheter with the use of IVUS does not affect the images to this extent
Catheter design	Minimum guide catheter: ${\mathrm{5F}}^{1}$	Minimum guide catheter: ${\mathrm{6F}}^{2}$	OCT catheter should be placed deeper in the target vessel and acquires images from the point of the mid to the proximal marker, whereas IVUS catheter can acquire images from its tip [19, 20]. This can put limitations to the use of OCT for lesions in the medial or distal part of a vessel
	Boston Scientific™ OPTICROSS HD: Three radiopaque markers: (i) distal marker, 5 mm from the tip of the catheter, with the transducer 20 mm from the tip of the catheter (ii) two proximal markers	Abbot™ Dragonfly OPTIS: Three radiopaque markers: (i) distal marker, at the tip of the catheter (ii) mid marker (position of the optical lens), 27 mm from the tip (iii) proximal marker, located 50 mm from the mid marker	
	Working length: 135 mm	Working length: 135 mm	
	Volcano Therapeutics™ Eagle Eye Platinum ST: Three radiopaque markers with 10 mm spacing and transducer 2.5 mm from the tip of the catheter	Fastview™ for OFDI: Three radiopaque markers and sensor position from tip 24 mm	
	Working length: 150 mm	Working length: 150 mm	
Tissue penetration (mm)	4–8	1–3	Ultrasound waves can penetrate deeper into tissue than light, so IVUS is better for the examination of all vessel walls, as well as vessel remodeling, something which could be problematic with OCT in cases of large luminal diameter or increased plaque burden [21]
Axial Resolution (μm)	100–150	10–20	Light enables more detailed visualization than ultrasound waves, so OCT achieves better visualization of vessel anatomy, intracoronary structures (i.e., plaque characteristics) and more reproducible images [21, 22]
Lateral Resolution (μm)	150–300	20–90	
Aqcuisition speed (mm/sec)	0.5	18–20	OCT images can be acquired in less time than with IVUS, which may play a crucial role, especially in complex clinical settings [18, 23]
Blood clearance	×	✓	IVUS does not require contrast, so it could be the preferable method in renal impairment [16]
3D-reconstruction	limited	✓	OCT has better resolution and can produce better 3D images, something exceptionally useful in complex clinical settings [24, 25, 26]

^1^The data are referred to the IVUS system of Boston Scientific™ OPTICROSS HD (which incorporates a mechanical catheter) and Volcano Therapeutics™ Eagle Eye Platinum ST (which incorporates a digital catheter). ^2^The data are referred to the OCT system of Abbot™ Dragonfly OPTIS and OFDI system Fastview™. IVUS, Intravascular Ultrasound; OCT, Optical Coherence Tomography.

## 4. Percutaneous Coronary Intervention (PCI) Guidance & Stent Optimization

Since intracoronary imaging modalities can assist to overcome several limitations of ICA in the catheterization laboratory, they can provide new insight into PCI and influence periprocedural decisions and outcomes. According to the latest American College of Cardiology (ACC)/American Heart Association (AHA) guidelines, IVUS has a class IIa recommendation in PCI guidance, especially in unprotected left main (LM) disease and complex coronary stenting [27]. Similarly, in the latest European Society of Cardiology (ESC)/European Association for Cardio-Thoracic Surgery (EACTS) guidelines IVUS has a class IIa recommendation in assessment and stent optimization of LM disease [28]. On the other hand, OCT in PCI guidance is considered by both sides of the Pacific Ocean a decent alternative of IVUS in stent optimization and was updated in class IIa recommendation [27, 28]. Regarding stent failure and the resulting problems leading to in-stent restenosis (ISR), the use of either IVUS or OCT is appraised reasonable.

### 4.1 IVUS versus Coronary Angiography

IVUS overcomes limitations of angiography in terms of vessel imaging, stent optimization and clinical outcomes of drug-eluting stent (DES) implantation and it is superior to angiography alone in simple and complex lesions.

In the DES era, intravascular imaging plays a crucial role in the context of coronary angioplasty. To the best of our knowledge, the largest meta-analysis comparing IVUS-guided with angiography-guided DES implantation included 4724 patients from 9 randomized trials, including the ULTIMATE trial [29]. It demonstrated that IVUS guidance was associated with lower risk of major adverse cardiovascular events (MACE) (5.4% vs. 9.0%, *p *< 0.001), cardiac death (0.6% vs. 1.2%, *p* = 0.03), target vessel revascularization (TVR) (3.5% vs. 6.1%, *p* = 0.001), target lesion revascularization (TLR) (3.1% vs. 5.2%, *p* = 0.001) and stent thrombosis (0.5% vs. 1.1%, *p* = 0.02) in a mean follow-up of 16.7 months. No difference was detected between the two groups regarding the incidence of all-cause death and myocardial infarction.

A previous meta-analysis enrolled 3 randomized and 14 observational trials with 26,503 patients and reported similar clinical outcomes [30]. Patients who had undergone IVUS-guided PCI had lower risk of having TLR (OR = 0.81, *p* = 0.046), death (OR = 0.61, *p *< 0.001), myocardial infarction (OR = 0.57, *p *< 0.001) and stent thrombosis (OR = 0.59, *p *< 0.001) compared to those who had undergone angiography-guided PCI. Moreover, the improved outcomes in the IVUS group are obvious in the meta-analysis of Elgendy *et al*. [31], which included 7 randomized trials and 3192 patients who underwent DES PCI. IVUS guidance was associated with lower risk of MACE (6.5% vs. 10.3%, *p *< 0.0001), cardiovascular death (0.5% vs. 1.2%, *p* = 0.05) and stent thrombosis (0.6% vs. 1.3%, *p* = 0.04). With this level of evidence, the superiority of IVUS over angiography in PCI guidance is unquestionable. Worth mentioning is that IVUS is, so far, the intravascular modality in which a decreased rate of death has been showed in meta-analyses.

### 4.2 OCT versus Coronary Angiography

Numerous studies have shown the benefits of OCT in catheterization procedures and its advantages in PCI guidance in contrast with plain angiography guidance [3, 4, 32]. The Centro per la Lotta contro l’Infarto-Optimisation of Percutaneous Coronary Intervention (CLI-OPCI) study was the first study to introduce OCT guidance in PCI [3]. Compared to angiographic guidance alone, angiography plus OCT demonstrated improved clinical outcomes in 1-year follow up, regarding MACE. Moreover, the use of OCT was not associated with major complications and led to additional interventions regarding management in almost 35% of patients.

Interestingly, OCT guidance has a significant impact in the procedural strategy of PCI. ILUMIEN I study was the first to demonstrate the influence of OCT on operators’ clinical decisions [32]. Operators’ decision-making was affected by pre- and post-PCI OCT images in 66% of cases, especially in more complex lesions. Pre-PCI OCT led to changes in stent length and diameter, whereas factors standing out by post-PCI OCT, as stent malapposition, stent underexpansion and edge dissection, resulted in additional in-stent post-dilation and/or new stent deployment. Moreover, OCT guidance seemed to improve clinical outcomes during and after the procedure -low 1 -month MACE rate.

Similarly, in the DOCTORS study, which is the first study to enroll patients with non-ST elevation acute coronary syndromes, the decision-making was affected by the OCT in half of the cases in the OCT-guided group [4]. OCT guidance succeeded superior hemodynamic parameters. Fractional flow reserve (FFR) values were higher in the OCT-guided group compared to the angiography guided group (0.94 ± 0.04 vs. 0.92 ± 0.05, *p* = 0.005). Despite the longer duration of the procedure and higher amount of contrast, OCT was not associated with increased incidence of complications during the procedure (i.e., MI, renal dysfunction). The main strength of the study is the optimization of stent expansion with the use of OCT.

One major concern is which approach would be the optimal treatment for cases with complex lesions and high-risk patients with several comorbidities. The ongoing ILUMIEN IV trial is sought to investigate the superiority of OCT-guided PCI compared to angiography-guided PCI in patients with complex lesions and/or diabetes mellitus [33]. Primary endpoints are the mean stent area (MSA) after revascularization and target vessel failure, which comprises cardiac death, target vessel myocardial infarction and ischemia-driven target vessel revascularization. The properties of OCT could improve acute PCI results and, thus, clinical outcomes in the future.

### 4.3 OCT versus IVUS versus Angiography

The first *in vivo* evaluation of optical frequency domain imaging (OFDI)—in comparison with IVUS was conducted by Okamura *et al*. [23]. OFDI imaging proved to be feasible and safe, with high reproducibility for both pre- and post-stenting areas. Unlike IVUS, OFDI systematically detected smaller minimal lumen diameter pre-stenting, which has been repeatedly described in literature, and detected vessel complications (thrombus, tissue prolapse, dissection, strut malapposition) at significantly higher rate.

One year later, Habara *et al*. [5] performed the first direct comparison of FD-OCT and IVUS in PCI guidance [5]. Interestingly, two major factors associated with stent thrombosis and restenosis, the minimal stent area (6.1 ± 2.2 mm vs. 7.1 ± 2.1 mm) and the MSA (7.5 ± 2.5 mm vs. 8.7 ± 2.4 mm) were constantly smaller with OCT than with IVUS (*p *< 0.05). Device and success rates were similar in the two groups, however, IVUS showed an advantage over OCT regarding vessel border visualization, stent expansion and the decrease in stent-edge plaque burden. Residual stenosis was detected more frequently with OCT but only at the proximal edge of the reference segment. No significant difference was detected between the two groups regarding stent apposition. In this study it was shown that FD-OCT emerges as a novel technique but there are still limitations in stent optimization.

A comparison of stent expansion (defined as the MSA divided by the mean of the proximal and distal reference lumen areas) with OCT or IVUS was attempted in the ILUMIEN II study, an observational study including 940 patients [6]. The results revealed comparable stent expansion in OCT and IVUS group (72.8% vs. 70.6%, *p* = 0.29). The detection of major stent malapposition, tissue protrusion or stent edge dissection was higher in the OCT group, but the difference was small. Nevertheless, further randomized trials are needed to compare IVUS and OCT in stent optimization.

The OPINION trial was a multicenter, non-inferiority trial comparing acute and mid-term outcomes of OCT-guided and IVUS-guided PCI [7]. The primary endpoint was target vessel failure defined as a composite of cardiac death, target-vessel related myocardial infarction, and ischemia-driven target vessel revascularization until 12 months after the PCI. The major secondary endpoint was angiographic binary restenosis at 8 months. There was a significantly smaller post-PCI MSA in the OFDI than the IVUS group, but the minimum lumen area (MLA) was comparable in both groups (*p* = 0.18). OFDI-guided stent implantation was associated with fewer proximal stent-edge hematomas, suggesting that it might be attributed to increased physiological stress through the stent expansion technique with IVUS. Moreover, fewer irregular protrusions were detected in the OFDI group. In the 8-month follow-up OFDI seemed to be related to lower neointimal proliferation—probably because of the less aggressive stent sizing—and higher percentage of uncovered struts, although there was no difference in the minimum lumen area between the two groups. OCT proved to be non-inferior to IVUS in the 12-month follow-up.

The first comparison of IVUS-guided versus OCT-guided versus angiography-guided PCI was attempted in the ILUMIEN III trial [8]. For the OCT-guidance an external elastic membrane (EEM)-based strategy in the proximal and distal reference segments was followed, to assess luminal diameter and lesion coverage. The EEM-based approach was defined as the smallest EEM area in the reference segment. Regarding MSA measurement, OCT was non-inferior to IVUS. Precisely, OCT guidance led to better stent expansion than IVUS guidance and to higher detection of edge dissection and stent malapposition than IVUS or angiography guidance.

The EEM-based strategy proved to be a safe method for stent sizing and provided several advantages over a lumen-guided approach, because it leads to the deployment of a larger stent size (~0.5 mm) and, subsequently, a larger luminal diameter without increasing the risk for post-procedural complications [12, 31]. OCT was superior in detection of suboptimal acute post-PCI outcomes compared to IVUS, except for cases of LM and ostial right coronary artery (RCA) lesions.

The most recent data by the iSIGHT (Optical Coherence Tomography Versus Intravascular Ultrasound and Angiography to Guide Percutaneous Coronary Interventions) trial elaborated the utility of OCT in PCI guidance [9]. In accordance with the ILUMIEN III, 151 patients were randomized to OCT-guided, IVUS-guided or angiography-guided-PCI. For both types of intravascular imaging an EEM-based protocol was applied for stent sizing, provided that EEM was visible in ≥180° of the vessel circumference. Otherwise, the maximal lumen diameter was used. OCT proved non inferior to IVUS (91.69 ± 15.75%, *p *< 0.001) and superior to angiography (90.53 ± 14.84%, *p* = 0.041), regarding stent expansion. The EEM-based sizing was delineated as of great importance in stent optimization.

Both intravascular imaging modalities have given new perspectives in PCI guidance. Both demonstrate a great potential in pre- and post-PCI clinical setting, with OCT emerging as a valuable tool in the assessment of PCI short- and long-term results with more precision than IVUS. On the other hand, IVUS is, so far, the intravascular modality most studied and with meta-analyses including death as a factor. Until today, OCT has proven to be non-inferior to IVUS, hence more data from future studies are needed to establish the one over the other in each clinical setting. Studies comparing OCT with IVUS and angiography in PCI guidance as well as ongoing studies are presented in Tables 1,3.

**Table 3.** **Ongoing studies of OCT- versus IVUS- versus angiography-guidance in PCI**.

Trial name or principal investigator	Estimated study completion date	N	Study design	Primary endpoint(s)
OCTIVUS (NCT03394079)	2028	1448	Randomized, non-inferiority, OCT vs. IVUS	TVF at 1 year
ILUMIEN IV (NCT03507777)	2022	2690	Randomized, OCT vs. ICA in high-risk patients and lesions	Post-PCI MSA measured by OCT TVF up to 2 years
RENOVATE (NCT03381872)	2022	1620	Randomized, IVUS vs. ICA in complex clinical settings	TVF at 1 year
Chen *et al*. (NCT03574636)	2025	375	Randomized, OCT vs. IVUS vs. QCA in moderate-to-severe calcifications	In-stent loss at 13 months

OCT, Optical Coherence Tomography; IVUS, Intravascular Ultrasound; PCI, Percutaneous Coronary Intervention; OCTIVUS, Optical Coherence Tomography Versus Intravascular Ultrasound Guided Percutaneous Coronary Intervention; TVF, Target Vessel Failure; ILUMIEN IV, Optical coherence tomography-guided coronary stent implantation compared to angiography; MSA, Minimum Stent Area; RENOVATE, Intravascular imaging-versus angiography-guided Percutaneous Coronary Intervention for complex Coronary Artery Disease; QCA, Quantitative Coronary Angiography.

### 4.4 LM Coronary Artery Disease

The anatomical location of LM lesions poses difficulties to the accurate evaluation with plain angiography [34]. This is particularly challenging in the presence of intermediate LM stenosis, which is not an uncommon phenomenon and where angiographic measurements cannot be so reliable. Certain pre-specified criteria for the cut-off lumen diameter both before and after PCI have been proposed. Park *et al*. [35] presented an IVUS-derived MLA of ≤4.5 ${\mathrm{mm}}^{2}$ as a useful index of an FFR ≤0.80 in functionally significant LM lesions. Fassa *et al*. [36] showed that deferral of PCI in 131 patients with an IVUS- measured MLA of ≥7.5 ${\mathrm{mm}}^{2}$ is a safe option with favorable clinical outcomes. Results from the LITRO study revealed that an MLA of 6 ${\mathrm{mm}}^{2}$ as a safe cut-off point for deferring PCI with favorable outcomes in 2-year follou-up.

Kang *et al*. [37] used IVUS to assess post-PCI MSA in LM lesions. 11.4% of patients demonstrated in-stent restenosis (ISR) in the 9-month follow up. The small MSA, defined by certain criteria on segmental basis and considering all areas of the bifurcation, including the polygon of confluence (POC), was a predictive factor of ISR. This segmental evaluation of the four parts, in which the LM was divided, was the novelty of this study. ISR was detected in almost half of the cases with stent under-expansion. This was accompanied with significantly higher rate of MACE in 2 years (90.2 ± 2.6% vs. 98.1 ± 0.9% at 2 years, *p *< 0.001). Stent malapposition was not related to increased rate of ISR and/or MACE. Similarly, the EXCEL trial provided a post-PCI MSA cut-off in LM lesions for Caucasians showed a strong correlation of an MSA of 4.4–8.7 ${\mathrm{mm}}^{2}$ with adverse events in 3-year follow up [38].

As addressed in the meta-analysis of Wang *et al*. [34], which included 4592 patients and compared IVUS-guided with angiography-guided drug-eluting stenting of LM coronary artery, IVUS demonstrated a clear benefit regarding hard endpoints: IVUS-guided LM coronary artery stenting reduced the risk of MACE, all-cause death, cardiac death, MI and stent thrombosis. As limitations of this meta-analysis could be considered the fact that the great majority of the included studies were observational and that IVUS guidance did not reduce TVR and TLR. In the latest ACC/AHA and ESC/EACS guidelines IVUS has a class IIa recommendation in the assessment of LM lesions [27, 28].

On the other side, OCT has not yet been incorporated in the ESC/EACS guidelines for LM angioplasty, whereas it is considered as a reasonable alternative of IVUS in the ACC/AHA guidelines, except of cases with ostial lesions. Because of the large diameter of LM coronary artery and the need for complete blood transposition, OCT is thought to be unsuitable for the assessment of LM lesions. Burzotta *et al*. [39] showed that FD-OCT may be a feasible and effective imaging modality for non-ostial LM lesions. Images can be obtained by applying special manipulation [39]. FD-OCT seems unsuitable for lesions of the proximal segment of the LM due to artifacts, but it effectively demonstrates the other LM segments and, thus, the bifurcation, which is the most commonly diseased site.

The LEMON study was a recent pilot study, that investigated whether patients with mid-LM coronary artery or distal-LM coronary artery disease will benefit from OCT-guided PCI based on a pre-specified protocol [40]. Stent sizing was based on external elastic lamina (EEL). OCT proved to be a safe and feasible method for PCI-guidance in 86% of cases and led to modification in operator’s strategy in 26% of cases, despite the adequate results in angiography. Exceptionally noticeable are the benefits of intracoronary imaging in more complex and high-risk lesions. However, the LEMON criteria did not include vessel anatomical characteristics and a specific analysis of the POC, which might influence stent expansion estimations. The percentage of optimal stent expansion was higher than in ILUMIEN III and DOCTORS trials, in which stent expansion was, also, high. OCT proved to be an effective modality for mid- or distal but not ostial lesions [39, 40, 41]. Nevertheless, the Telescope®(Medtronic Cardiovascular, Santa Rosa, CA, USA)-guiding extension catheter with its special technical characteristics demonstrated good visualization of aorto-ostial lesions, which remain a challenge in OCT-guided PCI [42].

### 4.5 Bifurcations

Bifurcation lesions account for 15–20% lesions in PCI procedures [43]. They comprise a demanding group of lesions, because of their complex morphological and physiological features and IVUS is considered as a useful tool for the invasive cardiologist. The latest ESC/EACTS guidelines on myocardial revascularization recommend IVUS-guided revascularization with class IIa for patients with coronary bifurcation lesions [28].

IVUS has demonstrated good results in bifurcation PCI procedures. In the meta-analysis of Yang *et al*. [43] IVUS-guided revascularization was associated with better short- and long-term clinical outcomes compared to angiography-guided revascularization. The risk for myocardial infarction and TLR or TVR was lower in the IVUS than in the angiography group (2.7% vs. 15.5% and 1.9% vs. 3.7%, respectively), which explains the lower risk of MACE in the IVUS group during the short-term follow-up and the risk for cardiac death in the long-term follow-up.

The important elements in decision-making regarding bifurcation lesions include anatomical and plaque characteristics, the presence of side branch ostial stenosis, the bifurcation angle, the length of the proximal side branch lesion and the distal side branch lesion diameter [44]. OCT is an excellent method for evaluating plaque features and can effectively assess the rest elements as well. Whereas all bifurcation segments can be adequately evaluated by OCT, the low penetration of light through lipid-rich plaque poses obstacles in visualization of the true lumen size, defined by the EEM. This has led to the general use of the media-to media diameter approach instead.

For each OCT round, about 10–15 mL of contrast are required, so the common practice for the visualization of the side branch is to perform one OCT examination before stent implantation and one after stent optimization, which is based on pre-stenting OCT images [44]. When 2 stents are required, the assessment of the side branch is of utmost importance but the angiographic assessment alone or the assessment only of the mother vessel by OCT is insufficient. OCT (especially 3D-OCT) is particularly useful for the guidance and optimization of side branch rewiring, and the evaluation of devices in bifurcation lesions and PCI results.

Ongoing studies are awaited to elaborate the role of intracoronary imaging in PCI guidance of bifurcations. Studies of OCT are about to drastically change our clinical practice. The OCTOBER trial (NCT03171311) aims to evaluate the clinical outcomes of OCT-guided PCI in bifurcations. Similarly, ILUMIEN IV trial is a large, multicenter, superiority trial which compares short- and long-term clinical outcomes of OCT-guided PCI versus angiography-guided PCI in high-risk patients and lesions, including bifurcations [4]. Regarding stent optimization, not only comparison but, also, combination has been studied: the BOOM (Bifurcation and Ostial Optical coherence Mapping) technique is a novel method, currently being evaluated for the precise mapping of the ostial side-branch segment, using OCT and angiography co-registration [45]. On the other hand, the IMPROVE trial (NCT04221815) is sought to evaluate the clinical outcomes and cost-effectiveness of IVUS-guided PCI in complex lesions.

### 4.6 Bioresorbable Scaffolds

Intracoronary visualization should be considered for the optimization during the implantation of bioresorbable scaffolds (BRS), because of their thick and wide struts. Nevertheless, limited data are available regarding the use of IVUS and OCT in PCI using BRS. Studies so far have demonstrated a rather favorable effect of OCT in PCI guidance with BRS. In their registry Caiazzo *et al*. [46] reported that there was a need for changing strategy in almost half of the patients treated with OCT-guided bioresorbable scaffolds implantation. Moreover, the examination of 45 patients included in the ABSORB trial with 20 MHz IVUS and OCT showed a clear advantage of OCT both in qualitative and quantitative parameters [47]. The two modalities had low agreement in terms of lesion, frame and strut assessment. OCT demonstrated superior ability to detect incomplete scaffold apposition, side branch struts, dissections and protrusions and an excellent overall reproducibility compared to 20 MHz IVUS.

Later investigations of BRS with IVUS with even higher resolution did not manage to establish it as a preferable method for PCI guidance in this setting. In a comparative study of 361 struts, as referenced to OCT assessments, the novel 60 MHz IVUS (axial resolution <40 μm) showed superiority over 40 MHz IVUS in terms of strut evaluation [48]. Still, the novel 60 MHz IVUS did not prove to perform better than OCT [49].

In treatment with BRS the so-called PSP (prepare the lesion, sizing appropriately, and post-dilation) criteria have been proposed for stent optimization and the avoidance of adverse cardiac events [50]. In the context of PSP criteria, OCT has a great potential in stent optimization [51]. But since there are no randomized trials with intravascular techniques in BRS implantation and bioresorbable scaffolds are only recommended for clinical trials, more studies are needed to shed light on the differential impact of IVUS and OCT in stent optimization, and their acute and future outcomes.

### 4.7 Slow-Flow & No-Reflow Phenomena

Intracoronary imaging could play an important role in prediction of slow flow and no-reflow phenomena and, thus, perioperative complications [52, 53, 54]. A pre-PCI IVUS examination of 687 atherosclerotic coronary plaques revealed, that lesion with echo signal attenuation and higher lipid core or microcalcification were strongly associated with post-PCI no-reflow [55]. Ultrasound attenuation with longitudinal length ≥5 mm has been associated with higher incidence of no-reflow in ST-Elevation Myocardial Infarction (STEMI) patients [53]. Predictive factors of these phenomena seem to be lesions characterized by large necrotic core or in acute plaque rupture, or Thin-Cap Fibroatheroma (TCFA) [53, 55]. Yamamoto *et al*. [56] delineated the possibly important role of IVUS in detecting high risk patients for slow-flow with LM-acute coronary syndrome (ACS). Factors predicting slow flow were the vessel diameter and the vessel area (defined as the EEM area).

Patients with ACS caused by plaque rupture are in greater risk for slow-flow and no-reflow phenomena. A retrospective analysis of 145 STEMI patients both with OCT and IVUS revealed occurrence of no-reflow in 40% of them [57]. The combination of OCT and IVUS highlighted the OCT-measured lipid index and the IVUS-measured plaque burden as two key factors for predicting no reflow in this group of patients. To the best of our knowledge, there is no other trial combining these two intravascular imaging modalities. Similarly, there are no randomized trials comparing OCT and IVUS in terms of slow-flow and no-reflow phenomena, which offers a broad field of investigation for risk factors associated with these phenomena.

### 4.8 Chronic Total Occlusions (CTO)

IVUS has an established role in CTO PCI. The most frequent cause of failure in CTO recanalization is inability of the guide wire to cross the lesion [58]. In a single center series of IVUS-guided stumpless wiring of CTOs, Ryan *et al. * [58] reported a success rate of 77% of cases and no major complications requiring intervention. IVUS has been proposed as a safe and effective tool in guiding the reverse CART approach. Dai *et al*. [59] examined the role of IVUS in reverse CART technique: IVUS successfully determined the appropriate balloon size and calcium-free zones with a success rate of 95.9%. 10.2% of cases presented major adverse events but without significant clinical outcome.

Although the IVUS guidance has not been proven superior to the conventional angiography guidance in CTO PCI in reducing MACE, it is associated with a lower risk of TLR, especially in long lesions, MI and stent thrombosis [60, 61]. On the other hand, OCT is currently being studied in the assessment of post-PCI procedural and anatomical results (stent apposition, coverage and endothelization) [62, 63].

### 4.9 Long-Term Clinical Outcomes

Intracoronary imaging is useful in determining predictors of long-term adverse events. Stent expansion and size, calcification and/or attenuation of a plaque are independent predictors of stent edge dissection [64]. Since geographical miss—defined as a significantly diseased segment or (balloon) injured segment not treated by stent—is linked to major adverse effects (TVR, MI, stent failure), intracoronary imaging could be of importance in detecting complications [64, 65, 66]. In an IVUS study, dissections associated with lumen diameter <5 ${\mathrm{mm}}^{2}$, with a length >3 mm and an arc >60° were related to greater risk for TLR [64]. Smaller luminal diameter and inflow/outflow disease (residual stenosis, dissection) were associated with early stent thrombosis after PCI for acute MI [67].

Favorable effects of IVUS guidance in PCI were, also, documented in more complex clinical settings. In the meta-analysis of Shin *et al*. [66], which included 3 randomized trials and 2345 patients with long lesions or chronic total occlusions (CTO), there was lower risk of MACE in the IVUS-guidance group compared to the angiography-guidance group in a mean follow-up of 1.4 years (0.4% vs. 1.2%, *p* = 0.040). Similarly, in the meta-analysis of Bavishi *et al*. [68] with 8 randomized trials and 3276 patients being included, a significant reduction of MACE (RR: 0.64, *p* = 0.0001), TLR (RR: 0.62, *p* = 0.004) and TVR (RR: 0.60, *p* = 0.007) rates was documented in the group of IVUS-guided PCI in 1 year. Of exceptional importance was the IVUS-guided PCI in long lesions, in diabetic patients and in patients suffering acute coronary syndrome.

On the other hand, OCT-guidance seems to positively affect decision-making—and, consequently, results—in PCI [3, 32]. It can effectively detect stent underexpansion, uncovered or malapposed struts, neointimal proliferation and major evaginations [69, 70]. Stent-related MI is associated with stent thrombosis, with or without restenosis [71]. Even though the in-stent thrombotic process after bare metal stent (BMS) and DES implantation is different, OCT proved to be a useful modality for definition of the special characteristics of each process and for the study of stent-related clinical events. Interestingly, MLA <4.5 ${\mathrm{mm}}^{2}$ and distal edge dissection >200 μm are high-risk characteristics for adverse events [72].

There are limited data from direct comparison of IVUS and OCT with respect to clinical endpoints. The currently running OCTIVUS trial (NCT03394079) is sought to compare safety and efficacy of IVUS-guided and OCT-guided revascularization in terms of target vessel failure (cardiac death, target-vessel myocardial infarction, or ischemia-driven target-vessel revascularization) at 1 year. A broad spectrum of PCI population was enrolled in this study which has already completed recruitment. Both non-inferiority and superiority of OCT will be evaluated. ILUMIEN III and OPINION trial proved non-inferiority of OCT, but they did not have the power to compare relevant clinical outcomes. OCT has more benefits compared to IVUS because of its higher resolution, however, more data are needed. In the context of further optimization there should be a careful consideration of potential benefits (i.e., in avoiding stent underexpansion) and risks (i.e., perforation).

## 5. Plaque Evaluation

Intracoronary imaging, by giving details of the plaque, helps in the determination of the cause of an ACS. The most predominant substrates for ACS are plaque rupture, plaque erosion, calcified nodule and spontaneous coronary artery dissection (SCAD), with the plaque rupture being the most common [73]. Rupture and erosion of atherosclerotic plaques have different pathophysiological patterns that are related to different outcomes [74]. Plaque rupture is associated with fibrous cap discontinuity at a site of a lipid-rich plaque, red blood cell-rich red thrombus and is more often seen in STEMI. On the other hand, plaque erosion is associated with intact fibrous cap with more smooth muscle cells, platelet-rich white thrombus, detachment of endothelial cells and is more often seen in Non-ST Elevation Myocardial Infarction (NSTEMI).

According to pathology studies the prevalence of plaque erosion in sudden cardiac death cases is 25–40% [74, 75, 76, 77]. Kubo *et al*. [77] evaluated lesions in 30 patients with MI using OCT, IVUS and angiography and reported an incidence of plaque erosion of 23%, 0% and 3%, respectively. Jia *et al. * [74] examined patients with OCT using a different detection algorithm and found that 31% of cases with ACS were attributed to plaque erosion. As a result, plaque erosion seems to be a frequent underlying mechanism for MI, which is underestimated during conventional ICA and it can be found only with the use of OCT.

IVUS has been combined with computational methods for the examination of biomechanical characteristics of vulnerable plaques [78]. Vulnerable plaques develop more often in proximal or bifurcation coronary segments, due to the increased turbulence of blood flow and the increased shear stress at these parts [79, 80, 81]. Plaque rupture occurs when shear stress within the plaque exceeds fibrous cap shear stress. Finite element analysis (FEA) is a computational method to calculate plaque structural stress (PSS). This analysis is useful to the prediction of plaque rupture. PSS was found to be higher in culprit lesion causing ACS compared to such lesions in patients with stable CAD. PSS is positively correlated to lumen diameter and negatively correlated to plaque burden [82]. Furthermore, it was higher in plaques with necrotic core area ≥10% and lower when dense calcium area ≥10% was present [83].

Analysis of IVUS radiofrequency (IVUS-RF) data provides more accurate and reproducible images than conventional IVUS [84]. It is a useful modality in the evaluation of atherosclerotic plaque composition and morphology and, thus, in the detection of vulnerable plaques [85]. Several studies delineated the role of IVUS-RF in the evaluation of plaque burden in high-risk patients and in cardiovascular risk stratifications [84, 85, 86, 87].

However, OCT seems to be the most appropriate imaging modality to identify predisposing factors/facts leading to ACS, due to its superior resolution [74]. OCT is an efficient method to detect the culprit lesion in ACS [72, 87]. The properties of OCT help to differentiate even among types of plaque erosion (definite, probable with/without luminal thrombus) [87]. OCT can distinguish between rupture-prone and erosion-prone plaques. In the EROSION study it was proposed that, although both plaque rupture and plaque erosion are similarly treated in the common practice, conservative treatment with antithrombotic therapy alone, without stent implantation, could be an option for plaque erosion [88]. Similarly, Amabile *et al*. [89] reported, that optimal medical treatment reduces the plaque burden, and this reduction can be effectively imaged with OCT.

Concerning future complications, OCT contributes to the detection of high-risk plaque characteristics that lead to adverse clinical outcomes. The CLIMA study demonstrated that minimal lumen area (MLA) <3.5 mm, lipid arc circumferential extension >180°, low fibrous cap thickness and OCT-defined macrophages were correlated to greater risk for MACE [90]. According to bibliography, patients with plaque erosion are at greater risk for suffering distal embolization, residual incidence of MI and stroke [90, 91, 92]. However, plaque erosion is related to better myocardial perfusion in patients with STEMI and lower risk of no-reflow [87, 93]. On the other hand, patients with plaque rupture run greater risk of MACE [94]. Furthermore, OCT contributes to the differentiation among plaque rupture, coronary thrombus and TCFA or the diagnosis of SCAD as a potential substrate for MI with non-obstructive coronary arteries (MINOCA) [95]. However, OCT seems inadequate to visualize plaque erosion *per se*; the diagnosis is based on the absence of finding of ruptured fibrous cap [96].

Moreover, calcification of coronary arteries increases the complexity of PCI procedures. Calcified lesions in IVUS appear as hyperechoic regions with acoustic shadowing. Because ultrasound waves cannot penetrate calcium, its extent is quantified by measuring its thickness, area or volume [97].

The presence of calcification in coronary arteries cannot be precisely evaluated by angiography and it may adversely influence PCI results, as it is a major cause of stent underexpansion [41, 44]. Thus, the role of intracoronary imaging could be crucial for optimizing the results in such patients. Calcifications in OCT appear as low-intensity regions with clearly demarcated calcified tissue borders [98].

When compared to angiography, IVUS enables higher detection of calcium. In a study of 1155 lesions, angiography detected calcium in 38% of them, whereas IVUS in 73% of them (*p* = 0.0001) [99]. Even though angiography can satisfactorily identify moderately calcified lesions, its sensitivity falls in milder degrees of calcification. On the other hand, IVUS demonstrated high sensitivity in calcifications. Recently, Liu *et al*. [100] proposed an IVUS-based calcium scoring system for the automated quantification of calcium. This framework was based on the on the A-line profile of manually annotated data from 35 pullbacks from the 3 most frequently used IVUS systems and displayed an accuracy of approximately 0.9, suggesting it could be of great importance in the image-guided interventional procedures.

Similarly, an OCT-based calcium scoring system has been developed to predict stent underexpansion [41]. According to the OCT-based calcium scoring criteria, lesions with calcium burden with maximum thickness >0.5 mm, length >5 mm and maximum angle >180°, are at higher risk for stent underexpansion, indicating that these lesions may benefit from pre-PCI plaque modification. Intravascular imaging could be a key feature in this procedure. Kobayashi *et al*. [64] strongly suggest OCT-guided rotational atherectomy (RA) for the treatment of calcified coronary lesions. They compared OCT-guided with IVUS-guided RA and reported significantly larger burr size and percent stent expansion in the OCT-guided RA group. Although a lower rate of TLR in one year in the OCT-guided compared to the IVUS-guided RA group was reported, it was not statistically significant.

An ongoing three-arm trial is sought to compare OCT-guided with IVUS-guided with QCA-guided stent implantation in moderate-to-severe calcified lesions (NCT03574636). The primary endpoint is the in-stent late loss, which is the difference between the minimal lumen diameter immediately post-PCI and the minimal lumen diameter as measured by angiography 13 months post-PCI. All in all, although both OCT and IVUS are useful in assessment of calcium, the high-intensity reflection of ultrasound waves in IVUS can put obstacles in assessment of calcium thickness [41, 96, 98]. On the other hand, OCT is better in thickness measurement, especially in superficial calcium deposits. To date, the use of OCT in very thick or deep calcium deposits is limited.

## 6. Intracoronary Imaging and FFR Assessment

Physiology assessment modalities have an established role in the detection of hemodynamically significant coronary lesions and, thus, their application affects decision-making in the catheterization laboratory [101, 102]. Several studies have been conducted either comparing or combining FFR with IVUS or OCT. Acomparativestudy of 167 consecutive patients with intermediate lesions who underwent either IVUS-guided or FFR-guided PCI demonstrated comparably favorable clinical outcomes in 1-year follow up [103]. Statistically significant difference was observed when PCI was performed; in the IVUS-guided group PCI rate was higher (RR = 0.02), suggesting that FFR-guidance might reduce the decision for intervention in such lesions. A recent meta-analysis of 16 randomized control trials and 17 propensity score weight-matched studies comparing IVUS-guided versus OCT-guided versus FFR-guided versus angiography-guided PCI showed analogous clinical outcomes among IVUS, OCT and FFR [104]. The setting in which both IVUS and OCT demonstrated more favorable outcomes than FFR was the subsequent MI in patients with previous ACS. Nevertheless, both intravascular imaging and functional modalities performed better than angiography alone, addressing the need for more extensive use of both in PCI guidance.

So far OCT has displayed moderate results considering hemodynamic assessment. In the ILUMIEN I trial a pre- and post-PCI combination of OCT and FFR was used [32]. Post-PCI FFR was lower (*p* = 0.003) in the group with unsatisfactory pre-PCI and post-PCI OCT results, but this difference was not detected among the groups after optimization. Furthermore, in a propensity matched analysis of patients who have undergone FFR-based PCI or OCT-based PCI the latter had similarly favorable outcomes [105]. There was a significantly lower incidence of TLR in the 25-month follow up in the OCT-guidance group compared to the FFR-guidance group (4.1% vs. 14.2%, *p <* 0.01). However, there was no significant reduction of MACE and all-cause mortality.

In a single-center randomized trial which compared FFR-guidance and OCT-guidance in angiographically moderate lesions there was a reduced incidence of MACE and significant angina in the OCT-guidance group compared to the FFR-guidance group (8% vs. 14.8%, *p* = 0.048) [106]. Pawlowski *et al*. [107] demonstrated a correlation of FFR and OCT in intermediate lesions in terms of MLA. The OCT threshold, which best correlated to hemodynamically significant FFR values, was MLA <2.05 ${\mathrm{mm}}^{2}$, addressing the possibility of using OCT along with FFR to assess hemodynamically significant lesions. Similar results regarding OCT-derived MLA were reported by Tomaniak *et al*. [104]. Although, they reported a moderate correlation of OCT-derived MLA and lesion length with FFR values, there was no difference in terms of plaque morphological characteristics between the hemodynamically significant and hemodynamically non-significant lesions, as assessed by FFR.

A combination of OCT and IVUS with FFR has been attempted through computational processing. The objective was to acquire both functional and anatomical information [104, 106, 107, 108]. Since these approaches are based on the quality of the 3D-imaging, OCT-based FFR is probably more accurate than IVUS-based FFR [109]. Still there is no head-to-head comparison of them, so more clinical findings are required.

## 7. Future Perspectives

**Fig. 3.**
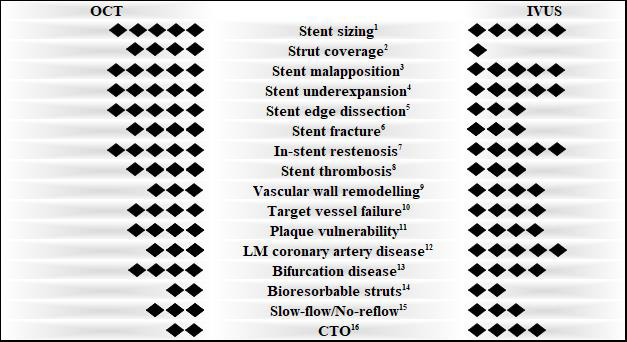
**Evidence-based use of intravascular imaging**. The ability of OCT and IVUS to evaluate specific factors in PCI is depicted as following: 5 rhombi = highly recommended, 4 rhombi = recommended, 3 rhombi = feasible, 2 rhombi = under investigation, 1 rhombus = not feasible. ^1,3,4,5,6,7,12^ High recommendation based on the latest guidelines [27, 28]. ^2^ For early-stage post-PCI [7, 8]. ^8^ [5, 38, 71]. ^9^ [21, 69]. ^10^ [7, 10]. ^11^ [74, 78, 88]. ^13^ [28, 43, 44]. ^14^ [47, 48, 49]. ^15^ [53, 55, 57]. ^16^ [60, 61, 62, 63]. PCI, Percutaneous Coronary Intervention; OCT, Optical Coherence Tomography; IVUS, Intravascular Ultrasound; LM, Left Main; CTO, Chronic Total Occlusion.

While evidence regarding the use of standalone OCT or IVUS is multiplying and establishes their role in different aspects of PCI (Fig. 3, Ref. [5, 7, 8, 10, 21, 27, 28, 38, 43, 44, 47, 48, 49, 53, 55, 57, 60, 61, 62, 63, 69, 71, 74, 78, 88]), multimodality imaging has, also, been attempted with positive results. Co-registration of OCT and IVUS combines the advantages of the two methods and demonstrates higher accuracy in plaque characterization than each method demonstrates alone [110, 111]. Olender *et al*. [112] describe the first co-registration of IVUS and OCT with the contribution of artificial intelligence (AI). Virtual Histology (VH)-IVUS provided the lesion characteristics and severity, while OCT provided a more detailed assessment of the lumen and the lesion. Through an AI-based system training, excellent synthetic OCT images could be produced based on the VH-IVUS images inserted in the system, paving the way of the deployment of sophisticated computational methods in the evaluation of intracoronary environment. However, co-registration of these modalities requires more time and 2 separate pullbacks, which impedes its broad application.

To surpass this problematic point, the development of hybrid systems has been attempted. The first dual-modality probes, combining OCT and IVUS that have been developed were tested on rabbit aortas [113, 114, 115]. The prototype had several limitations (e.g., large diameter, increased noise in IVUS images), so Yin *et al*. [116] introduced a novel combined miniature probe, with an outer diameter of 0.69 mm, capable of fitting in a 4-French sheath. This was achieved by rearranging the OCT probe and the IVUS transducer (sequential arrangement). In 2013 Li *et al*. [117] developed a new hybrid system with co-planar arrangement, which offered more accuracy in the simultaneous co-registration of OCT and IVUS images than sequential arrangement. Validation of these methods in human coronary arteries *in vitro* revealed good tissue and plaque characterization. In 2013 a variant OCT-IVUS imaging catheter was introduced, with back-to-back arrangement and higher frame rate than the previous ones (20 fps) and two years later a more advanced model enabled a frame rate of 72 fps [118, 119].

In 2018 Sheth *et al*. [120] performed the first-in-man application of hybrid OCT-IVUS catheter, enhancing the synergistic role of these modalities in plaque characterization and coronary interventions. The Novasight Hybrid System was developed by COVANI Medical Inc (Toronto, Canada) and researchers at the University of Toronto, with 3.3 catheter shaft, frame rate on hybrid mode of 100 fps and co-linear arrangement, which allows visualization of the vessel simultaneously with both modalities [121]. In a prospective, observational trial the Novasight system demonstrated efficacy and feasibility for both diagnostic purposes and in PCI guidance [122]. Hybrid image acquisition affected decision-making, highlighting the complementary role of the two modalities. Likewise, a Dual Sensor hybrid IVUS-OCT system, merging IVUS with OFDI, was developed by Terumo (Tokyo, Japan), with 3.2 catheter shaft, frame rate on hybrid mode of 100–160 fps and sequential arrangement. Fusion of OCT and IVUS images is also available.

Further data are needed to elaborate the use of fusion imaging in the intracoronary environment. There is a broad spectrum of phenomena regarding coronary plaques (vulnerability, plaque rupture, plaque erosion), in which research with hybrid methods can be really promising. Previous studies have demonstrated a clear advantage of combination of OCT and IVUS for detecting TCFA in comparison with each modality alone [123, 124, 125]. Randomized trials are warranted to establish whether standalone OCT, or IVUS or multimodality imaging, such as OCT-IVUS approach, or OCT-near infrared autofluorescence approach will have clinical effect in the assessment of necrotic core and intraplaque hemorrhage [126, 127]. Similarly, there is a broad field of investigation for IVUS and OCT combination in shear stress evaluation, where 3D-reconstruction and multimodality imaging play an important role [128, 129]. In the complex environment of multivessel coronary artery disease, IVUS-guided PCI has a class IIa recommendation in the latest guidelines [27, 28]. Whether hybrid methods could facilitate the procedure, needs to be studied.

One last major aspect is cost: IVUS seems to be a cost-effective method, still it has not been incorporated in daily practice [130]. Since standalone OCT and hybrid systems are more expensive and relatively time-consuming, should they be treated as luxuries or effective ways should be found to incorporate them in the catheterization laboratories?

## 8. Conclusions

Intravascular imaging brought a new insight in CAD. Indisputably, both OCT and IVUS techniques add significant information in the pathophysiology of lesions and their characteristics, which is valuable for their optimal treatment. This is reflected directly on the better outcome of the patients. Many studies have shed light on the use OCT and IVUS in PCI guidance. In addition, both OCT and IVUS are feasible and safe. For all these reasons, the use of intravascular imaging should be used more and more and be integrated in the daily practice of the interventional cardiologist. Nevertheless, the selection of imaging modality should be based on the clinical setting, the diseased segment, previous angiographic images, lesion characteristics and operator’s familiarity with each modality.
